# Supplementary material for: Temperature-Dependent Optical Properties of Perovskite Quantum Dots with Mixed-A-Cations
Source: Micromachines (Basel). 2022 Mar 17;13(3):457. doi: 10.3390/mi13030457 (PMC8955971; doi:10.3390/mi13030457)

Supporting Information

Temperature-dependent optical properties of perovskite quantum dots with mixed-A cations

Xiaoli Zhang[1], Lei Hu[1], Weijia Duan[1], Guojie Chen[2], and Bingfeng Fan[2,*] Weiren Zhao [1,*]

[1] Guangdong Provincial Key Laboratory of Information Photonics Technology, Guangdong University of Technology, Guangzhou, 510006, China

[2] Guangdong-Hongkong-Macao Joint Laboratory for Intelligent Micro-Nano Optoelectronic Technology, School of Physics and Optoelectronic Engineering, Foshan University, Foshan 528225, China

Figure S1. TEM images of perovskite QDs with mixed-cations (a) $FA_{0.9}Cs_{0.1}$, (b) $FA_{0.85}Cs_{0.15}$, (c) $FA_{0.8}Cs_{0.2}$.

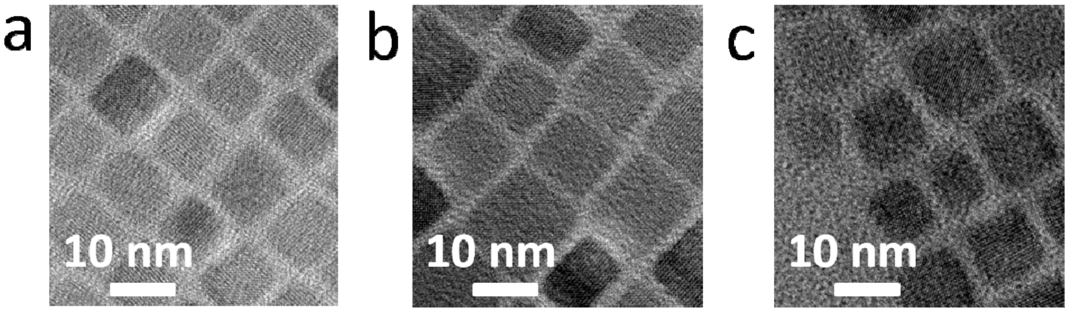

Figure S2. XRD patterns of perovskite QDs with mixed-cations (a) $FA_{0.9}Cs_{0.1}$, (b) $FA_{0.85}Cs_{0.15}$, (c) $FA_{0.8}Cs_{0.2}$.

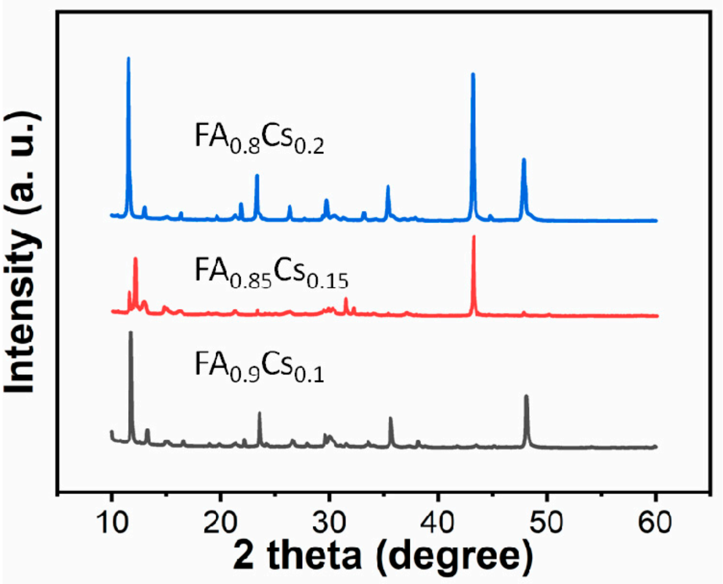

Supplement: Supplementary file 1 [file micromachines-13-00457-s001.zip › micromachines-1625850-supplementary.pdf]
